# Identification of three novel NHS mutations in families with Nance-Horan syndrome

**Published:** 2007-03-27

**Authors:** Kristen M. Huang, Junhua Wu, Simon P. Brooks, Alison J. Hardcastle, Richard Alan Lewis, Dwight Stambolian

**Affiliations:** 1 F.M. Kirby Center for Molecular Ophthalmology, University of Pennsylvania School of Medicine, Philadelphia, PA; 2 Division of Molecular and Cellular Neuroscience, Institute of Ophthalmology, University of London, London, UK; 3 Cullen Eye Institute, Baylor College of Medicine, Houston, TX

## Abstract

**Purpose:**

Nance-Horan Syndrome (NHS) is an infrequent and often overlooked X-linked disorder characterized by dense congenital cataracts, microphthalmia, and dental abnormalities. The syndrome is caused by mutations in the *NHS* gene, whose function is not known. The purpose of this study was to identify the frequency and distribution of *NHS* gene mutations and compare genotype with Nance-Horan phenotype in five North American NHS families.

**Methods:**

Genomic DNA was isolated from white blood cells from NHS patients and family members. The *NHS* gene coding region and its splice site donor and acceptor regions were amplified from genomic DNA by PCR, and the amplicons were sequenced directly.

**Results:**

We identified three unique *NHS* coding region mutations in these NHS families.

**Conclusions:**

This report extends the number of unique identified *NHS* mutations to 14.

## Introduction

Nance-Horan Syndrome (NHS; OMIM 302350) is an infrequent and often overlooked X-linked disorder characterized by dense congenital cataracts, microphthalmia, and microcornea [1-3]. Affected individuals develop dental anomalies which include serrated incisal edges and barrel-shaped teeth, in which the incisal edge and the gingival margin are narrower than the cervix of the tooth. Distinctive facial features include anteverted and often simplex pinnae and a long face with a narrow mandible and a narrow nasal bridge. Some affected males also display developmental delay [4,5]. The phenotype of female carriers is less severe but includes corneal diameters ranged midway between normal and the affected males, and Y-sutural opacities which may occasionally affect vision even at younger ages [6]. The variability among carriers presumably results from differentially random X-inactivation.

The gene for NHS was mapped by several investigators to the Xp22.13 region [5,7-11]. The *NHS* gene was isolated and confirmed by independent laboratories [12,13]. Two additional studies [14,15] identified unique *NHS* gene mutations in four other independent NHS families. Previously, we identified five North American families with the unifying phenotypic characteristics of NHS [6,16]. Here we report the analyses of *NHS* gene mutation of these five families, bringing the total number of unique mutations to fourteen.

## Methods

### Clinical examination

Families exhibiting X-linked congenital cataract and microcornea were ascertained through the Medical Retina and Ocular Genetics Service of the Cullen Eye Institute at Baylor College of Medicine in Houston and by regional and national referral. A detailed family history and a pedigree were obtained through personal interviews and corroborated by medical and ophthalmological examination. Each subject or the responsible adult signed a Consent for Participation which was approved by the Institutional Review Board for Human Subject Research of the Baylor College of Medicine and the corresponding committees at the Texas Children's Hospital and The Methodist Hospital, Houston.

Unambiguous distinctions between affected and normal male subjects were based on clinical criteria including head circumference, external ear length, the position of the pinnae, the shape of the helices, hand and finger lengths, dental anomalies, and ocular characteristics (Table 1). Blood samples were obtained by a single observer with no knowledge of linkage information. Detailed examination findings of individuals and family pedigrees have been published elsewhere [1,6,16,17].

**Table 1:** Clinical characteristics of NHS patients.

Family	Congenital cataract	Nystagmus	Microcornea	Microphthalmia	Dental anomalies	Mental retardation	Brachymetacarpalia	Dysmorphic facial features
XL-11	+	+	+	+	+	No	+	+
XL-56	+	+/-	+	+	+	No	+	+
XL-51	+	+	+	+	+	No	+	+
XL-116	+	+	+	+	+	No	+	+
XL-39	+	+	+	+	+	No	+	+

### Mutation screening and sequence analysis

Venous blood from family members established permanent EBV-transformed lymphoblastoid cultured cell lines with standard techniques [18]. Genomic DNA was isolated from EBV transformed cells using Qiagen kits, following the manufacturer's recommendations. Exons from the *NHS* gene were amplified from genomic DNA (100 ng) with primers that span intron-exon junctions and primers within single large exons (primer sequences listed in Table 2). This analysis included exon 3a which is variably expressed in human *NHS* transcripts [19]. PCR was performed with 0.5 U AmpliTaq gold polymerase (ABI, Foster City, CA), 10 mM Tris HCl, pH 8.3, 1.5 mM MgCl_2_, and 50 mu M each dATP, dTTP, dGTP, and dCTP in 50 mu l volume. Amplification was performed following these conditions: 94 °C for 5 min, followed by 35 cycles at 95 °C for 1 min, 55-60 °C for 30 s, and 72 °C for 1 min with a final extension at 72 °C for 7 min. PCR products were separated by gel electrophoresis, purified over gel filtration columns (Novagen, Madison, WI), and cloned into TOPO II vectors (Invitrogen, Carlsbad, CA), or sequenced directly as necessary on a 3900 DNA Sequencer (Applied Biosystems, Foster City, CA). For each mutation found in families XL116, XL11, and XL39, the presence of the mutation was also analyzed in two unaffected family members.

**Table 2:** Primer sequences used in PCR of genomic DNA.

**NHS1 exon**	**Primer sequence**
1	1F TATCCGGACTGCCAGATCGC
	1R GAGTAGTAAGGTGCAAGCTGC
2	2F GTTGGCCAAAAGCACAACTT
	2R GGTGTGTTGGGGCTGATG
3	3F ACTCCCAAGGGGAAAAGAGA
	3R TTCCTCAGCAGCAAGCATAG
	3aF ATACACTGTGTTGTGTGCACG
	3aR TCTGGACAGAGTGGGATAGG
4	4F TTCCTTTGTCCTAAGGGCCTA
	4R TGGTATTCTTAGCAGCACAGA
5	5F TGAGACCTATTTGTGGGTTGC
	5R TCTGTACTAGGCGGAGGAATG
6	6.1 F TCACTGTGCTTTCCATGTGC
	6.1 R ATGTGGCTGCTAAGGAGGAC
	6.15F GGCCTGCTCTCAACATCTTC
	6.2F GCCACATGGACCAGAAAGAT
	6.2R CAAGAGGCAGCTTCATTTCC
	6.3F CAGCACCTGCCTCACAGTT
	6.3R TCTTCAGACTTGTTGATGGACCT
	6.4 FAAGCAG AAC ACAGTAGG AG AAACA
	6.4R TCCTTCTGTGGGAAAAGCAC
	6.5F TCTCCCTTATTTAGAGGAAAGCA
	6.5R TACCAGGCACTTTGTCATGG
	6.6F GCAGTTGAGATGGGACCAGA
	6.6R ATTCCAGGAAGTGCCATGAG
7	7F TAGCGTGCTGGGTAACTTCC
	7R GGGGCAAAACCTTTGTTGTA
8	8F GTGAGATGTTTGCCCCATTT
	8R GTAAGGGTTTTGGCCTTTGC
	8.1 F TGAGATGTTTGCCCCATTTT
	8.1 R TGGCAGACATGCGGTAACTA
	8.2F AAGAAAGGCAGTCGCTCAGA
	8.2R GTAAGGGTTTTGGCCTTTG

## Results & Discussion

**Figure 1:**
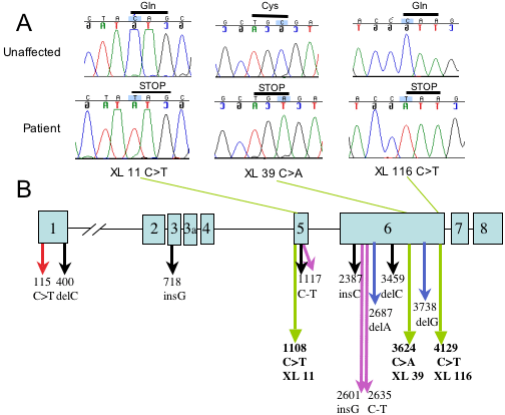
*NHS* mutation detection and localization. **A**: The identification of *NHS* nonsense mutations in three NHS families. Chromatograms from one affected and one unaffected individual are shown for each family. Above each tracing the base change is highlighted and the predicted stop codon is indicated. **B**: The localization of the fourteen reported *NHS* mutations within the *NHS* gene: red arrow [14]; black arrows [12]; green arrows (this study); purple arrows [15]; and blue arrows [13].

In family XL-116, we identified a C >T nonsense mutation in all tested affected individuals, at nucleotide 4129 of the 4893 bp human *NHS* cDNA. This mutation introduces a putative premature stop codon at the end of exon 6 and would result in a truncated protein that lacks a small part of exon 6 and all of exons 7 and 8. Affected individuals in family XL-39 carry a C >A nonsense mutation that introduces a putative premature stop codon at nucleotide 3624. This mutation falls in the middle of exon 6 and results in a truncated protein lacking part of exon 6 and exons 7 and 8. Family XL-11 displays a C >T nonsense mutation at nucleotide 1108. This mutation occurs in exon 5 and predicts a truncated protein lacking part of exon 5 and all of exons 6, 7, and 8. None of these mutations was observed in any unaffected family members. No coding region or splice site alterations were identified in families XL-56 and XL-51. These mutation results are presented as Figure 1 and Table 3.

**Table 3:** Summary of mutation in the *NHS* gene.

**Reference**	**Family**	**Exon**	**Genomic mutation**	**Predicted protein change**
[12]	1	6	c.2387insC	p.S797 fsX
	2	6	c.3459delC	p.L1154fsX28
	3*	5	c.1117C>T	P.R378X
	4	3	c.718insG	p.E240fsX36
	5**	1	c.400delC	p.R134fsX61
	6	-	No mutation	
[13]	1	6	c.3738-3739delTG	p.C1246-A1247fsX15
	2**	1	c.400delC	p.R134fsX61
	3	6	c.2687delA	p.Q896fsX110
	CRX	-	No mutation	
[14]	1	1	C.1150T	p.Q39X
[15]	P8598	IVS 3-2	c.853-2 A>G	Splice site change
	P20079	6	c.2601insG	p.K868E fsX5
	P21540*	5	c.1117C>T	P.R378X
	P24486	6	c.2635C>T	p.R879X
This study	XL 39	6	c.3624C>A	P.C1208X
	XL 116	6	c.4129C>T	P.Q1358X
	XL 11	5	c.1108C>T	P.Q370X
	XL 51	-	No mutation	
	XL 56	-	No mutation	

The asterisk indicates that family three from Burdon et al. [12] is unrelated to family P21450 [15] and the double asterisk indicates that family five from Burdon et al. [12] is related to family 2 from Brooks et al. [13].

Our analyses are consistent with previous reports that showed no correlation between the *NHS* genotype and the severity of NHS signs among affected family members [12,15]. For example, patients carrying the C >A mutation at bp 3624 present with typical NHS ocular and dental features [15]. In contrast, patients with a TG deletion at the neighboring positions 3738-3739 have bilateral cleft palate and the classical NHS signs [13] (family 3). Similarly, Ramprasad et al. [14] and Burdon et al. [12] identified two different *NHS* mutations within exon 1. However, the reported phenotype in patients bearing a mutation at nucleotide 400 is more severe than those with a mutation at nucleotide 115. A detailed structure-function analysis of the NHS protein should reconcile the phenotypic differences in the several NHS families.

Our study includes two families with no identified *NHS* mutations in the coding region. This is consistent with previous studies [12,13] and suggests the possibility of regulatory or intronic mutations in these families. Interestingly, the equivalent mouse *Xcat* mutant, which is the model for human NHS, does not display coding region mutations but does carry a large insertion mutation within the first intron of the mouse homolog of *NHS* (*Nhs1*) [20]. This insertion alters the expression of the *Nhs1* transcript and the Nhs protein in *Xcat* mice. Perhaps the NHS families who do not display *NHS* coding region mutations carry alterations in intron 1 structure. It will be interesting to explore this possibility further through the use of cytogenetic and gene expression studies on the *NHS* transformed cell lines. This study identifies additional *NHS* gene mutations in NHS families and validates the model that the *NHS* gene is required for proper eye development in humans. In addition, this work is consistent with the role of mouse *Nhs1* in cataract formation. Based on the phenotype of NHS patients, the *NHS* gene has many specialized functions in several different tissues. Functional studies on the NHS protein should help illuminate its role in eye, ear, tooth, and digit development.
